# Emergence of carbapenem-resistant and colistin-susceptible *Enterobacter cloacae* complex co-harboring *bla*_IMP-1_ and *mcr-9* in Japan

**DOI:** 10.1186/s12879-020-05021-7

**Published:** 2020-04-16

**Authors:** Pegah Kananizadeh, Satoshi Oshiro, Shin Watanabe, Shu Iwata, Kyoko Kuwahara-Arai, Masahiro Shimojima, Miho Ogawa, Tatsuya Tada, Teruo Kirikae

**Affiliations:** 1 Department of Microbiology, Juntendo University Graduate School of Medicine, 2-1-1 Hongo, Bunkyo-ku, Tokyo, 113-8421 Japan; 2 BML, Inc Department of Microbiology, Kawagoe, Saitama, Japan

**Keywords:** *Enterobacter cloacae* complex, *E. Xiangfangensis*, *E. Asburiae*, *Mcr-9*, *bla*_IMP-1_, Carbapenem resistance, Colistin resistance

## Abstract

**Background:**

The spread of *Enterobacteriaceae* producing both carbapenemases and Mcr, encoded by plasmid-mediated colistin resistance genes, has become a serious public health problem worldwide. This study describes three clinical isolates of *Enterobacter cloacae* complex co-harboring *bla*_IMP-1_ and *mcr-9* that were resistant to carbapenem but susceptible to colistin.

**Methods:**

Thirty-two clinical isolates of *E. cloacae* complex non-susceptible to carbapenems were obtained from patients at 14 hospitals in Japan. Their minimum inhibitory concentrations (MICs) were determined by broth microdilution methods and E-tests. Their entire genomes were sequenced by MiSeq and MinION methods. Multilocus sequence types were determined and a phylogenetic tree constructed by single nucleotide polymorphism (SNP) alignment of whole genome sequencing data.

**Results:**

All 32 isolates showed MICs of ≥2 μg/ml for imipenem and/or meropenem. Whole-genome analysis revealed that all these isolates harbored *bla*_IMP-1_, with three also harboring *mcr-9*. These three isolates showed low MICs of 0.125 μg/ml for colistin. In two of these isolates, *bla*_IMP-1_ and *mcr-9* were present on two separate plasmids, of sizes 62 kb and 280/290 kb, respectively. These two isolates did not possess a *qseBC* gene encoding a two-component system, which is thought to regulate the expression of *mcr-9*. In the third isolate, however, both *bla*_IMP-1_ and *mcr-9* were present on the chromosome.

**Conclusion:**

The *mcr-9* is silently distributed among carbapenem-resistant *E. cloacae* complex isolates, of which are emerging in hospitals in Japan. To our knowledge, this is the first report of isolates of *E. cloacae* complex harboring both *bla*_IMP-1_ and *mcr-9* in Japan.

## Background

The emergence of carbapenemase-producing *Enterobacteriaceae* (CPE) has become a serious problem in medical settings worldwide [1]. The most frequently detected and globally widespread carbapenemase produced by CPE between the Asian countries are the class B metallo-β-lactamases (MBLs), which include IMP-type, NDM-type, and VIM-type MBLs [2].

Because of the emergence of multidrug-resistant Gram-negative pathogens and the lack of new antibiotics with efficient activities, colistin, a polymyxin-type antibiotic, has been the last resort used to treat CPE infections [3, 4]. Bacteria acquire colistin resistance through chromosomal mutation(s) or plasmid transfer [5]. Chromosome-mediated colistin resistance results from mutation(s) or deletion(s) of two component systems, such as *phoPQ* and *pmrAB*, altering the structure of lipopolysaccharides [6, 7]. For example, colistin resistance has been associated with modification of the lipid A moiety in lipopolysaccharide, such as by the addition of 4-amino-4-deoxy-L-arabinose (L-Ara-4 N) and phosphoethanolamine (PEtN) to the anionic phosphate groups of lipid A. These additions reduce the anionic charges on lipid A and its affinity to the cationic colistin, inhibiting membrane destruction resulting from the binding of colistin to lipid A, followed by cell death [8].

To date, various types of plasmid-mediated mobilized colistin-resistance genes, *mcr*, have been identified, including *mcr-1* to *mcr-9*, with several, including *mcr*-1, − 2, − 3, − 4, and − 6, shown to have PEtN transferase activity [6]. The *mcr-1* gene was initially detected in isolates of *Escherichia coli* and *Klebsiella pneumoniae* obtained from humans and animals in 2015 in China [9], and *mcr-9* was initially identified in a clinical isolate of the colistin-susceptible bacterium, *Salmonella enterica* serotype *typhimurium*. The amino acid and nucleotide sequences of *mcr-9* are closest to those of *mcr-3* with similarities of 64.5 and 99.5%, respectively [10]. In this study, *mcr-9* was detected in 335 genomes in multiple genera of *Enterobacteriaceae*. The analysis of *mcr-9* promoter region in these genomes showed conserved regions which is likely a recognition sequence for transcription regulator, suggesting that other factors might be involved in full-expression of *mcr-9.* Of the 335 genomes, 65 had at least one plasmid replicon indicating that *mcr-9* can be found extrachromosomally in different species of *Enterobacteriaceae* [10].

Isolates of *E. cloacae* complex resistant to both carbapenem and colistin have been reported in several countries, including China [11, 12], France [13], India [14], the USA [15, 16] and Vietnam [17]. One of these, an isolate of *E. cloacae* complex (*Enterobacter hormaechies*) co-harboring *bla*_VIM-4_ and *mcr-9*, was first reported in the United States in 2019 [16]. In addition, a colistin-resistant *E. hormaechei* isolate producing both MCR-9 and NDM-1 was isolated from a patient in China with bloodstream infection in 2019 [11]. This emergence of colistin resistance, particularly in CPE, may result in significant clinical and public health concerns [18, 19].

The study describes three clinical isolates of *E. cloacae* complex that were resistant to carbapenem but susceptible to colistin. To our knowledge, this is the first report of isolates of *E. cloacae* complex harboring both *bla*_IMP-1_ and *mcr-9* in Japan.

## Methods

### Bacterial strains

Thirty-two clinical isolates of *E. cloacae* complex, each with minimum inhibitory concentrations (MICs) of ≥2 μg/ml for meropenem and/or imipenem, had been obtained from individual patients at 14 hospitals in eight prefectures throughout Japan from July to October 2018 by BML Biomedical Laboratories R&D Center (Kawagoe, Saitama, Japan).

### Drug susceptibility testing

The MICs of antibiotics were determined using a broth microdilution method according to the guidelines of the Clinical and Laboratory Standards Institute (CLSI) [20]. The MICs of colistin were also determined by a broth microdilution using cation-adjusted Muller Hinton broth and 96-well microtiter plates (Kohjin Bio, Co., Ltd. Saitama, Japan) according to the guidelines of the European Union Committee for Antimicrobial Susceptibility Testing (EUCAST) [21].

### Whole genome sequencing

DNA was extracted from each *E. cloacae* complex isolate using DNeasy Blood and Tissue kits (Qiagen, Tokyo, Japan). A Nextera XT DNA library was prepared from each extracted DNA sample. Each DNA library of was sequenced on the MiSeq system (Illumina) to obtain short reads with 300-bp paired-end reads. MiSeqRun was performed using Nextera XT Index Kit v2 and MiSeq Reagent Kit v3. DNA Libraries for MinION (Oxford Nanopore Technologies, Oxford, UK) were prepared from three isolates (A2483, A2504 and A2563) using Ligation Sequencing Kits 1D (SQK-LSK109) to yield long contigs. The long read generated by MinION were assembled using Canu v1.7.1 and polished with the short reads generated by MiSeq using Pilon v1.22. The nucleotide sequences of plasmids and chromosomes carrying *bla*_IMP-1_ and *mcr-*9 were compared with similar sequences using BLAST and visualized by In silico MolecularCloning. Ver.7 genomic edition (https://www.insilicobiology.co.jp/).

Bacterial species were identified by analyses of average nucleotide identity (ANI) [22] and digital DNA-DNA hybridization (dDDH) [23] of whole genome sequences. The seven type strains used as reference species included *Enterobacter asburiae* (ATCC35953^T^), *E. cloacae* (ATCC13047^T^), *E. hormaechei* (ATCC49162^T^), *Enterobacter kobei* (DSM13645^T^), *Enterobacter ludwigii* (EN-119^T^), *Enterobacter nimipressuralis* (DSM18955^T^) and *Enterobacter xiangfangensis* (LMG27195^T^). In silico multilocus sequence typing (MLST) was assigned by PUBMLST database (https://pubmlst.org/databases/). Acquired antibiotic resistance genes were identified using the ResFinder 3.2 tool (https://cge.cbs.dtu.dk/services/ResFinder/) from the Center for Genomic Epidemiology (CGE).

### Phylogenetic analysis based on SNPs

Single nucleotide polymorphisms (SNPs) in the 32 isolates were identified by aligning whole-genome sequencing data of these isolates with the genomic sequences of the *E. xiangfangensis* reference isolate LMG27195 (GenBank accession no. CP017183.3), using the CSI Phylogeny 1.4 tool (https://cge.cbs.dtu.dk/services/CSIPhylogeny/) from CGE. A phylogenetic tree was constructed using Fig Tree (version 1.4.4) and a maximum likelihood phylogenetic tree (http://tree.bio.ed.ac.uk/software/figtree/).

## Results

### Phenotypic and genotypic properties of carbapenem-non-susceptible isolates

#### Drug susceptibility of carbapenem-non-susceptible isolates

The MICs of the 32 clinical isolates of *E. cloacae* complex are shown in Table 1. All were susceptible to amikacin and colistin, but resistant to ceftazidime. Of these 32 isolates, 25 were resistant to aztreonam, 15 were resistant to ciprofloxacin, and 12 were resistant to tigecycline. Of the all 32 isolates, 28 isolates were resistant to imipenem and/or meropenem with MICs ≥4 μg/ml, whereas the remaining 4 were intermediate to imipenem and/or meropenem with MICs ≥2 μg/ml (Table S1). There are no isolates susceptible to both imipenem and meropenem (Table S1).

**Table 1:** MIC values of 32 clinical *E. cloacae* complex isolates

Antimicrobial Agents^a^	Breakpoint for resistance (μg/ml)	No. of resistant Isolates (%)	MIC data (μg/ml)		
			Range	MIC_50_	MIC_90_
Amikacin	≥64	0	0.5 to 4	1	2
Aztreonam	≥16	25 (78.1%)	< 0.25 to 256	32	256
Ceftazidime	≥16	32 (100%)	32 to > 512	256	> 512
Ciprofloxacin	≥4	15 (46.87%)	< 0.25 to 64	2	32
Colistin^b^	> 2	0	0.03 to 2	0.25	0.5
Imipenem	≥4	18 (56.25%)	< 0.25 to16	4	8
Meropenem	≥4	18 (56.25%)	0.5 to 16	4	16
Tigecycline^b^	> 0.5	12 (37.5%)	< 0.25 to 4	0.5	1

^a^Breakpoints for antimicrobial resistance were determined according to CLSI guidelines

^b^Breakpoint for Colistin and Tigecycline was determined according to EUCAST guidelines

#### Whole genome sequences of carbapenem-non-susceptible isolates

**Fig. 1:**
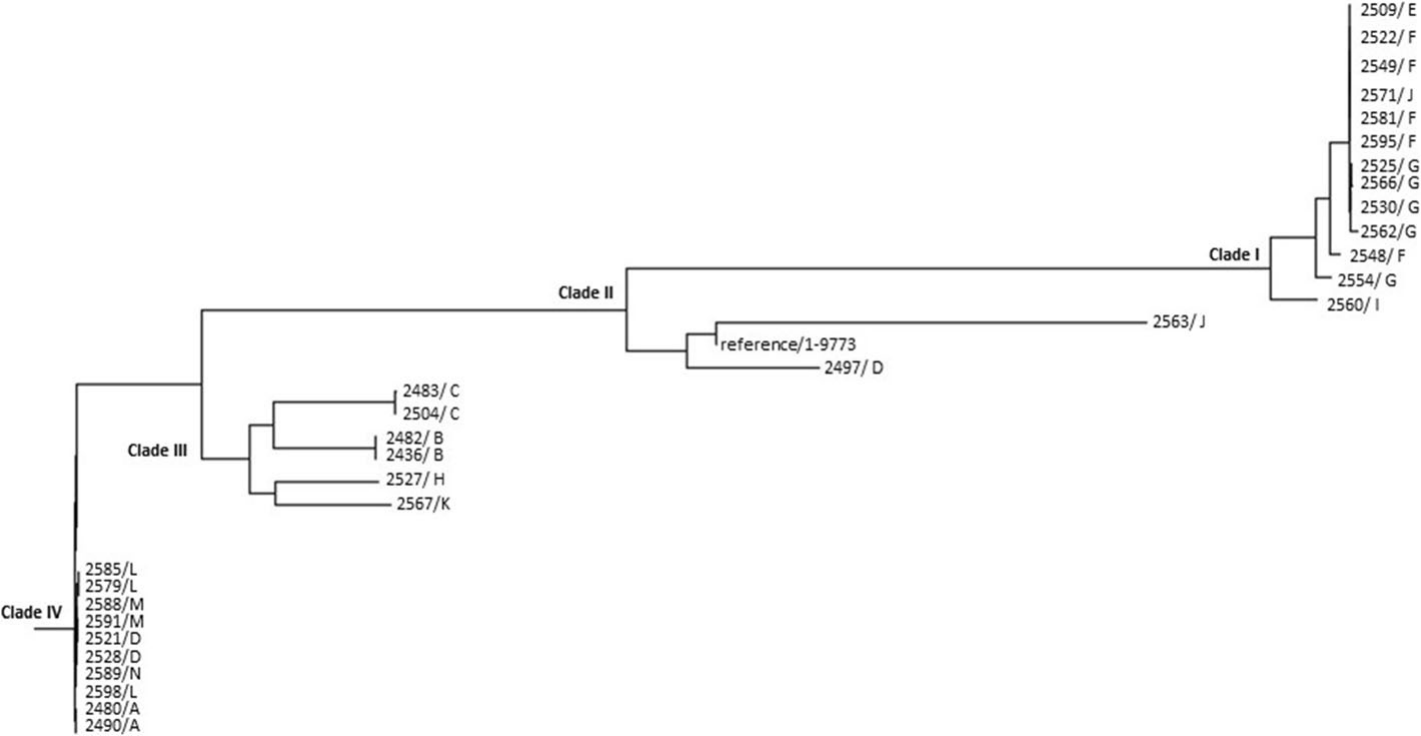
Phylogenetic tree of 32 isolates of *E. cloacae* complex obtained from 14 hospitals in eight prefectures throughout Japan. The phylogenetic tree was constructed by the maximum-likelihood method based on single nucleotide polymorphisms (SNP) in the core genome and aligned with *E. xiangfangensis* reference isolate LMG27195 (GenBank accession no. CP017183.3) (Isolates No./Hospital No.)

Whole genome sequencing of the 32 isolates of *E. cloacae* complex showed that, based on ANI and dDDH analyses, 31 were *E. xiangfangensis* and one was *E. asburiae*. MLST analysis revealed that 13 isolates (40.6%) belonged to sequence type (ST) 78; 10 (31.2%) to ST133; two each (6.3%) to ST175 and ST1196; and one each (3.1%) to ST62, ST93, ST418, and ST484. The ST for one isolate could not be determined because its housekeeping genes did not match those of current STs. A phylogenetic tree of these 32 isolates revealed four major clades, with clades I, II, III and IV consisting of 14, 2, 10 and 6 isolates, respectively (Fig. 1). Clade I consisted of isolates belonging to ST78 and the non-typeable isolate, clade II of isolates belonging to ST418 and ST484, clade III of isolates belonging to ST133 and clade IV of isolates belonging to ST1196, ST175, ST93 and ST62. These isolates harbored various genes associated with drug resistance (additional file: Table S1). All 32 isolates harbored *bla*_IMP-1_, with three also harboring *mcr-9* (Table S1).

### Phenotypic and genotypic properties of isolates harboring both *bla*_IMP-1_ and *mcr-9*

#### Bacterial identification and drug susceptibility

Of three isolates co-harboring *bla*_IMP-1_ and *mcr-9*, two, A2483 and A2504, were *E. xiangfangensis* and one, A2563, was *E. asburiae* (Table S1). The A2483 and A2504 strains obtained in a hospital belonged to ST1199, whereas the A2563 strain obtained in another hospital belonged to ST484. The two hospitals located in the same prefecture in Japan. The drug susceptibility profiles of the two *E. xiangfangensis* isolates were identical to each other, with both A2483 and A2504 being resistant to aztreonam, ceftazidime, imipenem, meropenem and tigecycline, and susceptible to amikacin, ciprofloxacin and colistin (Table 2). The *E. asburiae* isolate was resistant to ceftazidime and imipenem, had intermediate resistance to meropenem, but was susceptible to the other drugs tested including colistin (Table 2).

**Table 2:** Drug susceptibility profile of *E. cloacae* complex isolates co-harboring *mcr-9* and *bla*_IMP-1_

Isolates/Antimicrobial Agents^a^	MIC (μg/ml)							
	AMK	AZT	CAZ	CIP	CST^b^	IPM	MPM	TIG^b^
*E. xiangfangensis* A2483	1	128	> 512	1	0.125	4	8	1
*E. xiangfangensis* A2504	1	128	> 512	1	0.125	4	8	1
*E. asburiae* A2563	0.5	0.25>	128	0.5	0.125	8	2	0.5

^a^Breakpoints for antimicrobial resistance were determined according to CLSI guidelines

^b^Breakpoints for Colistin and Tigecycline was determined according to EUCAST guidelines

#### Whole genome sequences of isolates harboring both bla_IMP-1_ and mcr-9

As shown in Table 3, *E. xiangfangensis* A2483 contained a chromosome of 5,024,985 bp with a GC content of 55.24% and two plasmids of 61,594 bp and 288,696 bp, respectively. *E. xiangfangensis* A2504 contained a chromosome of 4,934,510 bp with a GC content of 54.70% and two plasmids of 61,594 bp and 276,927 bp, respectively. The whole genome sequences of A2483 were very close to those of A2504 with similarities of 100% (98% query coverage) on the chromosome, 100% for the 62-kbp plasmid and 100% (96% query coverage) for the 289-kbp plasmid. *E. asburiae* A2563 contained a chromosome of 4,934,510 bp with a GC content of 55.80% and one plasmid 115,246 bp in size (Table 3). In addition to *bla*_IMP-1_ and *mcr-9,* these isolates harbored several other genes associated with drug resistance, including *aac (6′)-IIc*, *bla*_ACT-6_, *bla*_ACT-7_, *fosA* and *sul1* (Table 3). *bla*_ACT_ genes are the intrinsic *AmpC* encoding genes of *Enterobacter cloacae* complex species.

**Table 3:** Genetic characterization of carbapenem-resistant and colistin non-resistant *E.cloacae* complex isolates coharboring *bla*_IMP-1_ and *mcr-9*

Isolates	Genetic contents	Plasmid type	Size (bp)	GC content	Antibiotic resistance genes
*E. xiangfangensis* A2483	chromosome		5,024,985	55.24%	*bla*_ACT- 7_*, fosA*
	plasmid	IncHI2	288,696	46.30%	*mcr-9*
	plasmid		61,594	47.40%	*aac(6′)-Iic, bla*_IMP-1_, *sul1*
*E. xiangfangensis* A2504	chromosome		4,934,510	54.70%	*bla*_ACT- 7,_*fosA*
	plasmid	IncHI2	276,927	46.30%	*mcr-9*
	plasmid		61,594	47.40%	*aac(6′)-Iic, bla*_IMP-1*,*_*sul1*
*E. asburiae* A2563	chromosome		4,808,368	55.80%	*bla*_ACT- 6*,*_*bla*_IMP-1*,*_*mcr-9, sul1*
	plasmid	IncFIB (pECLA)	115,246		

#### Location of mcr-9 and its genetic environments

**Fig. 2:**
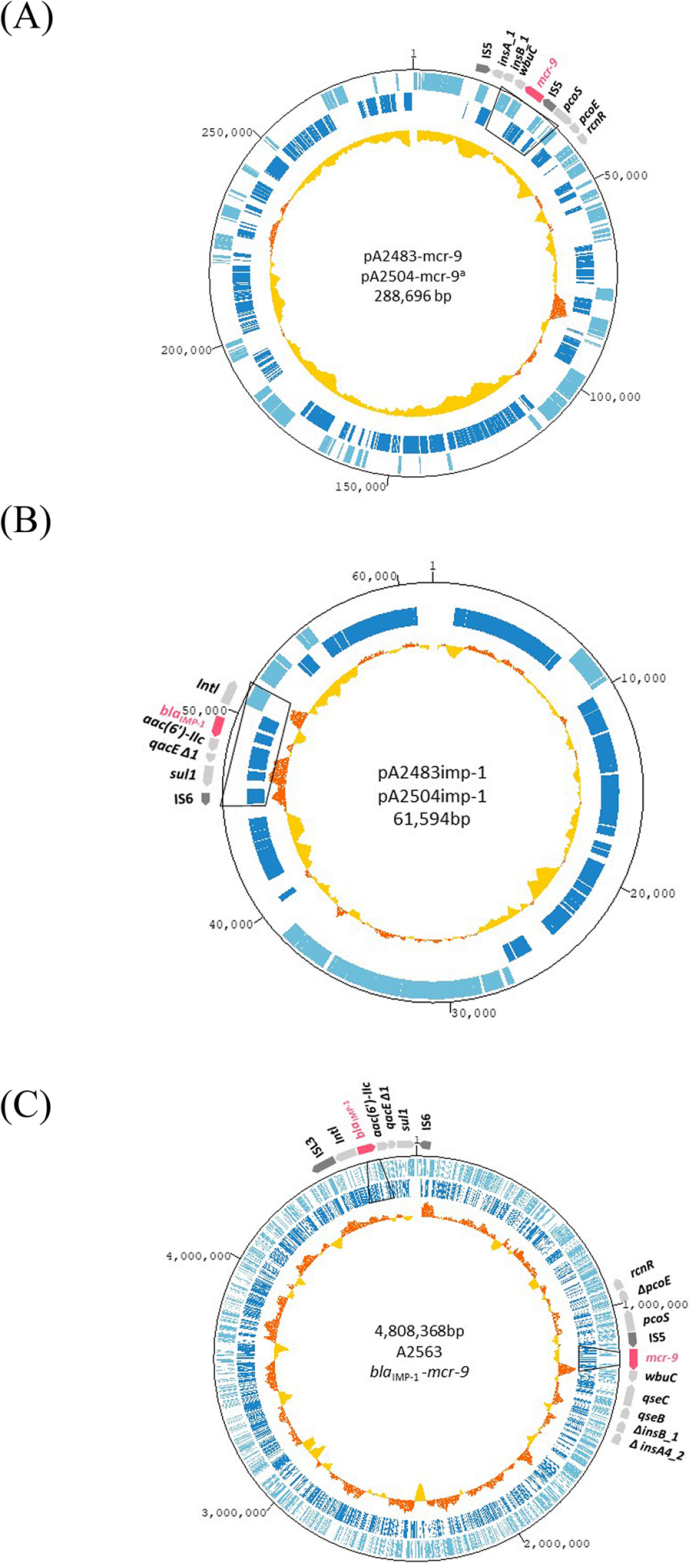
Circular structures of chromosome and plasmids harboring *bla*_IMP-1_ and *mcr-9*. **a** Map of the plasmids pA2483mcr-9 and pA2504mcr-9, showing that, relative to pA2483mcr-9, ^a^ pA2504-mcr-9 had a deletion of 11,770 bp from nt 146,310 to nt 158,080. **b** Map of the plasmids pA2483imp-1 and pA2504imp-1, showing that their structures were identical. **c** Map of the chromosome of A2563 coharboring *bla*_IMP-1_ and *mcr-9*. The two outermost circles show forward (light blue) and reverse (dark blue) genes and the innermost circle show GC content, with dark orange indicating above average and light orange indicating below average. Colored arrows indicate the positions and directions of antibiotic resistance genes (pink), surrounding genes (light grey) and insertion sequences (dark grey). Truncated genes are indicated by *∆*

The *mcr-9* gene was present on the 289-kpb IncHI2 plasmid of A2483 and the 277-kpb IncHI2 plasmid of A2504, but was present on the chromosome of A2563 (Table 3). The two plasmids harboring *mcr-9*, pA2483mcr-9 on A2483 and pA2504mcr-9 on A2504, had the same GC content of 46.30%, and contained open reading frames (ORFs) of 360 and 358, respectively (Fig. 2. (a)). The nucleotide sequences of these plasmids were identical to each other, except for a genetic region with 11,770 bp, from nucleotide (nt) 146,310 to nt 158,080, in the 277-kbp plasmid. The *mcr-9* gene on the chromosome of A2563 was detected at nt ~ 129 Mb.

**Fig. 3:**
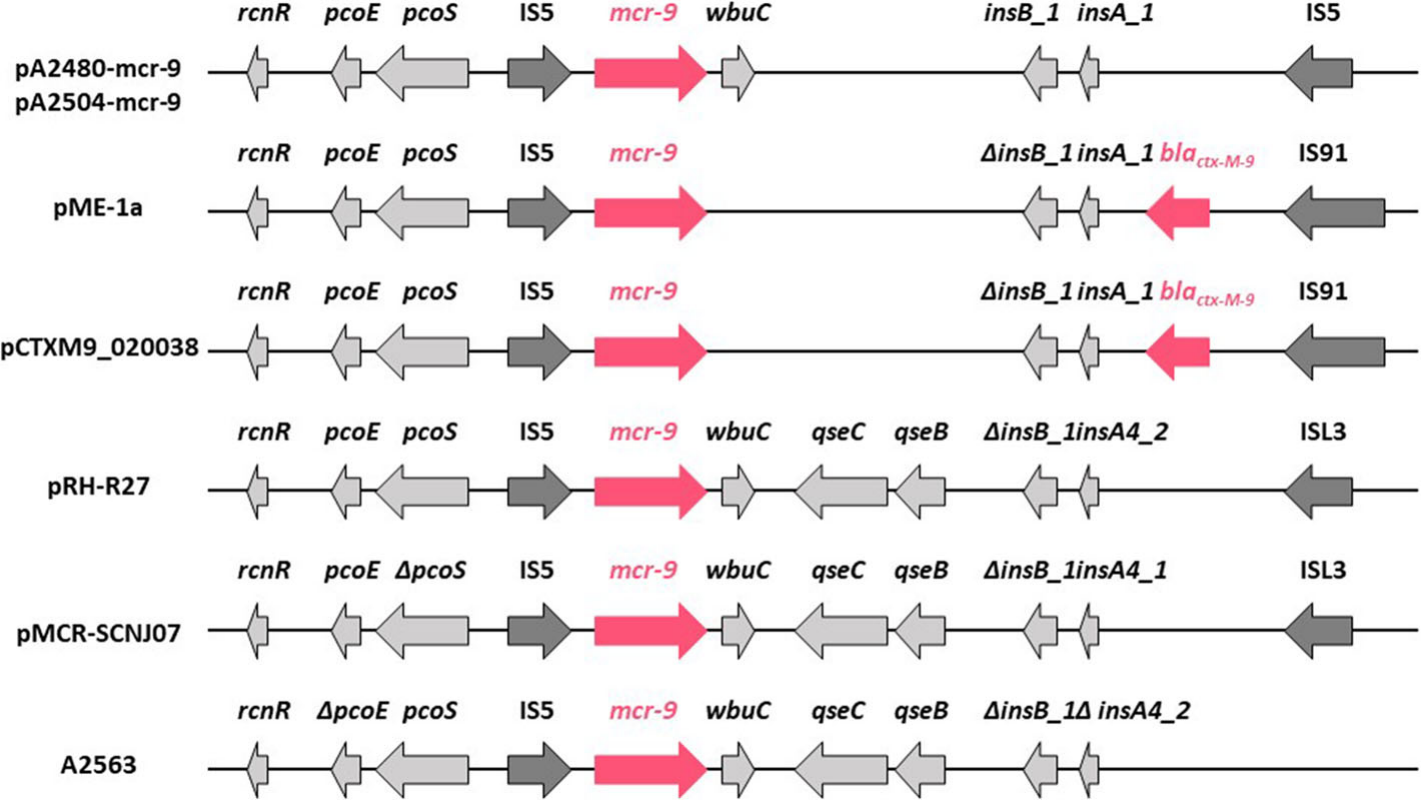
Linearized genetic environments of *mcr-9* harbored by IncHI2-type pA2483mcr-9, pA2504mcr-9 (this study), pME-1a (GenBank accession no. NZ_CP041734.1), pCTXM9_020038 (GenBank accession no. CP031724), pRH-R27 (GenBank accession no. LN555650), p707804-NDM (GenBank accession no. MH909331 and by the A2563 chromosome (this study). Colored arrows indicate positions and directions of antibiotic resistance genes (pink), surrounding genes (light grey) and insertion sequences (dark grey). Truncated genes are indicated by *∆*

The genetic environments of *mcr-9* in the A2483 and A2504 plasmids were identical to each other, with *mcr-9* located in a ~ 30 kb region surrounded by two insertion sequences encoding an IS5-like element (IS903 family transposase; Fig. 3). The region upstream of *mcr-9* included *rcnR* (encoding a Ni/Co-binding transcriptional repressor), *pcoS* (encoding a two-component sensor histidine kinase) and *pcoE* (encoding a copper-binding protein). The region downstream of *mcr-9* included *wbuC* (encoding a cupin fold metalloprotein) but no genes encoding the two-component system *qseC-qseB*, which has been associated with the expression of *mcr-9* [24]. Insertion sequences were not detected in the region downstream of *mcr-9* on the A2563 chromosome. The region upstream of *mcr-9* was *rcnR*-*pcoS*-Δ*pcoE*, whereas the region downstream of *mcr-9* was *wbuC*-*qseC-qseB* (Fig. 3). The A2483 and A2504 plasmids showed 83% query coverage and 99.97% identity to the IncHI2 plasmid, pME-1a (GenBank accession no. NZ_CP041734.1), in *E. hormaechei*, a strain isolated in 2019 from a pediatric inpatient in the USA (Fig. 3) [16]. Three IncHI2 plasmids were identified with similar sequences, pCTXM9_020038 from *E. hormaechei* isolated in China in 2018 (83% query and 99.97% identity; GenBank accession no. CP031724), pRH-R27 from *Salmonella enterica* Infantis in Germany in 2015 (82% query coverage and 99.99% identity; GenBank accession no. LN555650), and pMCR-SCNJ07 from *E. hormaechei* isolated in China in 2019 (80% query and 99.99% identity; GenBank accession no. MK933279) (Fig. 3).

#### Location of bla_IMP-1_ and its genetic environment

The *bla*_IMP-1_ gene was present on the 62-kbp plasmids of A2483 and A2504 and on the chromosome of A2563 (Table 3). The two plasmids harboring *bla*_IMP-1_, pA2483imp-1 from A2483 and pA2504imp-1 from A2504, had the same GC content of 47.40% and identical nucleotide sequences (Fig. 2 (b)). In these plasmids, *bla*_IMP-1_ was located in a class I integron containing *intl*-*bla*_IMP-1_-*aac (6′)-IIc*-*qacE∆1*-*sul1*. The *bla*_IMP-1_ gene on the chromosome of A2563 was present in the same class I integron (Fig. 2 (c)). The same class I integron containing *bla*_IMP-1_ and *aac (6′)-IIc* was detected in the bacteria *E. asburiae* NUH15_ECL035_1 (GenBank accession no AP019388.1), *Enterobacter cloacae* NUH15_ECL020 (GenBank accession no AP019386.1) and *Enterobacter asburiae* NUH12_ECL030 (GenBank accession no AP019383.1), all of which were isolated in Japan in 2019. The two pA2483imp-1 and pA2504imp-1 showed 84% query coverage and 97.75% identity with the plasmid pJJ1886_4 (GenBank accession no CP006788.1), which was detected in the USA and did not contain a class I integron or any other resistance genes [25].

## Discussion

The *mcr-9* gene may be silently spreading in *Enterobacteriaceae* throughout the world. The prevalence of *mcr-9* is unclear because this gene is not actually related to colistin resistance, as it may be silent or inducible in clinical isolates of *Enterobacteriaceae*. For example, an isolate of *E. hormechei* harboring *mcr-9* did not express its gene product [16]. This isolate was susceptible to colistin, likely because the two-component system genes *qseCB* were lacking from the region downstream of *mcr-9*. In contrast, another isolate of *E. hormechei* that harbored and expressed *mcr-9* was found to be resistant to colistin and to have the two-component system genes in the region downstream of *mcr-9* [11]. The expression of *mcr-9* is mediated by the two-component system QseCB and can be induced by subinhibitory concentrations of colistin [24]. At least 11 *mcr-9-*positive IncHI2 plasmids have been detected by Blast, with six having and five lacking the two-component system genes [11].

The two-component QseCB system, consisting of a sensor (qseC) and a response regulator (qseB), plays an essential role in the expression of *mcr-9* [24]. Our finding, that the isolate A2563 harbored *mcr-9* along with the two-component system genes *qseCB* but was susceptible to colistin suggests that other, as yet undetermined, genes or molecules may regulate *mcr-9* expression. The pA2480mcr-9 and pA2504mcr-9 had similar structures to those of pME-1a and pCTXM9_020038, as they lacked *qseCB*. This two-component system was transcribed as an operon, with the QseB promoter binding to low- and high-affinity binding sites located − 500 to − 10 bp at upstream of *qseB* [26]. The nucleotide sequence of this region in A2563 was 100% identical to that of the QseB promoter (− 500 to + 1 bp) in pMCR-SCNJ07, which confers resistance to colistin [11], suggesting that the QseB promoter in A2563 may be repressed by an as yet undetermined mechanism [26]. Four plasmids, pME-1a, pCTXM9_020038, pRH-R27 and pMCR-SCNJ07, had the conserved gene structure, *rcnR*-*pcoS*-*pcoE*-IS-5, upstream of *mcr-9*. Whereas, the chromosome of A2563 had the same conserved gene structure, but with a 53-bp deletion in *pcoE* (Δ*pcoE*), suggesting that the deleted region may be associated with *mcr-9* expression. Further studies are necessary to determine the mechanism for regulation of *mcr-9* expression in *Enterobacteriaceae.*

To our knowledge, it is the first report describing a bacterial isolate harboring *mcr-9* on its chromosome, indicating that *mcr-9* may have been inserted into the chromosome by mobile elements. Several *Enterobacteriaceae* isolates from animals and humans have reported the chromosomal location of *mcr-1* and *mcr-2* [27–33]. The *mcr-1* was detected on the chromosomes of two colistin-resistant *E. coli* strains isolated from swine in 2012 in China [28], and on the chromosome of an *E. coli* ST410 strain harboring *bla*_CTX-M-15_ isolated from a sample of turkey meat in 2013 in Germany [27]. The chromosomal integration of *mcr-1* was also detected in a clinical strain of *E. coli* ST156 harboring *bla*_NDM-5_ isolated from a bile sample in 2015 in China [29], in *E. coli* isolated from food production animals in 2011–2016 in Poland [32], and in *E. coli* isolated from veal calves in 2016 in the Netherlands [30]. Chromosomes carrying *mcr-1* were detected in *Enterobacteriaceae* from environmental water sources in 2017 in China [33]. Moreover, the *mcr-2* gene (*mcr-6.1*) was detected on the chromosome of a strain of *Moraxella* isolated from a pig in 2014–2015 in Great Britain [31]. These studies support the mobility characterization of *mcr* genes across different genetic elements and insertion of the plasmid-variant of *mcr* into chromosome could lead to higher prevalence of colistin resistance among *Enterobacteriaceae* specious.

The direct origin of the *mcr-9* on the chromosome of A2563 is unclear. However, the genetic environments of the *mcr-9* and *qseCB* genes in A2563 are similar to those of pMCR-SCNJ07 from *E. hormaechei* in China in 2019 (GenBank accession no. MK933279), pRH-R27 from *Salmonella enterica* Infantis in Germany in 2015 (GenBank accession no. LN555650), pT5282-mphA from *E. cloacae* in China in 2012 (GenBank accession no. KY270852), pN1863-HI2 from *E. cloacae* in China in 2017 (GenBank accession no. MF344583), pSE15-SA01028 from *S. enterica* subsp. *enterica* in Germany in 2018 (GenBank accession no. NZ_CP026661) and p707804-NDM from *Leclercia adecarboxylata* in China in 2018 (GenBank accession no. MH909331). These 7 strains carrying plasmids with *mcr-9* in China and Germany did not harbored *bla*_IMPs_, but *bla*_NDMs_ or *bla*_VIMs_ [11].

The plasmids pA2483imp-1 and pA2504imp-1 had the same backbone as the plasmid pJJ1886_4 (GenBank accession no CP006788.1), which had been isolated in the USA. The 55,956 bp plasmid pJJ1886_4, which was smaller in length than the 61,594 bp plasmids pA2483imp-1 and pA2504imp-1, lacked a class I integron carrying *bla*_IMP-1_ (*intl*-*bla*_IMP-1_-*aac (6′)-IIc*-*qacE ∆1*-*sul1)*. The *E. cloacae* EN3600 plasmid (GenBank accession no CP035638.1) carrying *bla*_IMP-8_ also had the same backbone as pJJ1886_4, with 83% coverage and 96.8% identity. These findings indicate that pJJ1886_4 has spread globally and captured drug-resistance genes and that this plasmid functions as a carrier of acquired drug-resistance genes*.*

## Conclusion

In conclusion, this study describes the characterization of the complete genomes of three clinically obtained isolates of carbapenem-resistant and colistin-susceptible *E. cloacae* complex harboring both *bla*_IMP-1_ and *mcr-9* from different hospitals in Japan. *Enterobacteriaceae* harboring both *bla*_IMP-1_ and *mcr-9* may become a healthcare problem, suggesting the need for steps to prevent their further dissemination.

## Supplementary information

**Additional file 1: Table S1.** Phenotypic and genotypic properties of 32 carbapenem-non-susceptible of *E. cloacae* complex isolates

## Data Availability

All the data necessary to reproduce the results can be found in the manuscript’s tables; the calculations can be reproduced using the manuscript’s appendixes. The genome sequence of strain A2563 has been deposited into GenBank under the accession number of AP022628. The complete nucleotide sequence of pA2483mcr-9, p2483imp-1, pA2504mcr-9, and p2504imp-1 have been deposited into GenBank under the accession numbers of LC532224, LC532225, LC532226 and LC532227, respectively.
